# Hydrogen-Assisted Brittle Fracture Behavior of Low Alloy 30CrMo Steel Based on the Combination of Experimental and Numerical Analyses

**DOI:** 10.3390/ma14133711

**Published:** 2021-07-02

**Authors:** Yunlong Li, Keshi Zhang, Damin Lu, Bin Zeng

**Affiliations:** 1 Key Laboratory of Disaster Prevent and Structural Safety, Guangxi Key Laboratory Disaster Prevent and Engineering Safety, College of Civil Engineering and Architecture, Guangxi University, Nanning 530004, China; zhangks@gxu.edu.cn (K.Z.); ludamin@st.gxu.edu.cn (D.L.); 1810401002@st.gxu.edu.cn (B.Z.); 2 College of Energy Engineering and Building Environment, Guilin University of Aerospace Technology, Guilin 541004, China

**Keywords:** hydrogen embrittlement, low alloy steel, cohesive zone modeling, hydrogen-enhanced decohesion, CTOD-R curve

## Abstract

Compact-tension (CT) specimens made of low alloy 30CrMo steels were hydrogen-charged, and then subjected to the fracture toughness test. The experimental results revealed that the higher crack propagation and the lower crack growth resistance (CTOD-R curve) are significantly noticeable with increasing hydrogen embrittlement (HE) indexes. Moreover, the transition in the microstructural fracture mechanism from ductile (microvoid coalescence (MVC)) without hydrogen to a mixed quasi-cleavage (QC) fracture and QC + intergranular (IG) fracture with hydrogen was observed. The hydrogen-enhanced decohesion (HEDE) mechanism was characterized as the dominant HE mechanism. According to the experimental testing, the coupled problem of stress field and hydrogen diffusion field with cohesive zone stress analysis was employed to simulate hydrogen-assisted brittle fracture behavior by using ABAQUS software. The trapezoidal traction-separation law (TSL) was adopted, and the initial TSL parameters from the best fit to the load-displacement and J-integral experimental curves without hydrogen were calibrated for the critical separation of 0.0393 mm and the cohesive strength of 2100 MPa. The HEDE was implemented through hydrogen influence in the TSL, and to estimate the initial hydrogen concentration based on matching numerical and experimental load-line displacement curves with hydrogen. The simulation results show that the general trend of the computational CTOD-R curves corresponding to initial hydrogen concentration is almost the same as that obtained from the experimental data but in full agreement, the computational CTOD values being slightly higher. Comparative analysis of numerical and experimental results shows that the coupled model can provide design and prediction to calculate hydrogen-assisted fracture behavior prior to extensive laboratory testing, provided that the material properties and properly calibrated TSL parameters are known.

## 1. Introduction

Hydrogen atoms penetrate into the interior of steel which can change the mechanical properties of steel, leading to the premature fracture of steel components. This phenomenon is called hydrogen embrittlement (HE) [1,2,3]. The degradation in metallic materials due to HE is a complex mechanism question which combines mechanical and physical–chemical standpoints. Previously, related research showed that the mechanistic characteristics of hydrogen-assisted fracture transformed from dimples ductile fracture (microvoid coalescence (MVC)) without hydrogen to brittle fracture (quasi-cleavage (QC) or intergranular (IG) fracture + transgranular (TG) fracture mechanism) under hydrogen-charged condition [4,5]. For the HE mechanism, some classical theories have been developed, and so far the following three theories have been widely accepted: a hydrogen-enhanced decohesion (HEDE) mechanism [6,7,8,9], in which it is assumed that hydrogen atoms entering the steel causes lattice expansion and reduces surface energy. The second theory is a hydrogen-enhanced localized plasticity (HELP) mechanism [10,11,12,13], which states that hydrogen atoms in steel enhance dislocation mobility, thus reducing apparent yield stress and promoting local plastic deformation under low stress or stress intensity factor. The third theory is an adsorption-induced dislocation emission (AIDE) mechanism [9,10,11,12,13,14], and it is assumed that hydrogen atoms promote dislocation emission and motion to reach critical conditions, and hydrogen-induced crack nucleation causes damage. Apparently, out of these three theories, no single theory can provide adequate explanation of the HE mechanism, thus it seems that different mechanical systems correspond to different theories [15,16,17]. It is generally believed that when the local hydrogen concentration (driven by the high hydrostatic pressure) in front of the notch tip or notch root exceeds a critical value, the materials’ strength decreases giving rise to crack initiation. Novak et al. [18] built a comprehensive micro-mechanical model based on physical statistics, and proposed a synergistic effect of HELP and HEDE on hydrogen-induced IG fractures in steels. Wasim et al. [19] developed a new model for hydrogen degradation of the K_Q_ of steel based on the fractography of the specimen, and confirmed that the simultaneous action of HELP + HEDE could be active depending on the local concentration of hydrogen in low carbon steel. Zafra et al. [20] studied the fracture toughness behavior of 42CrMo4 and 2.25Cr1Mo steels under low displacement rate under pre-charged hydrogen and obtained J−Δa curves of compact tension (CT) specimens. The results of microstructural study revealed the existence of tempered martensite, and the fracture surfaces exhibited a mixed mode IG and lath decohesion fracture, which revealed the HELP-mediated HEDE mechanism. They concluded that the HEDE was the final failure mechanism. The similar research results have also been found in previous scientific reports [21,22]. Although the role of the HELP mechanism cannot be ignored in various material-hydrogen systems, the HEDE mechanism can still play a dominant role in the research of HE for low alloy steel investigated in this study. Many recent studies affirmed that these steels (with yield stress of over 1000 MPa) exhibit traces of IG fracture mode, and it is usually believed that the critical grain-boundary (GB) hydrogen concentration weakens the cohesive strength of GB and leads to the GB separation following the HEDE mechanism [23,24].

Nowadays, numerical simulation is attracting progressively more attention as a method for HE modeling. Integration of the experimental data with numerical analysis can effectively provide improved comprehensive understanding of the interaction between hydrogen and steel. Numerical models have been explored and developed by Sofronis and McMeeking [25], Krom et al. [26] and Taha and Sofronis [27], who introduced the framework to allow the coupling between hydrogen diffusion and mechanical behavior. Furthermore, a coupled deformation-hydrogen diffusion-phase field fracture scheme of the finite element method (FEM) was introduced by Martínez-Pañeda et al. [28]. Cohesive zone modeling (CZM) is attracting significant research interest as an approach for failure analysis [29,30]. For instance, Sung [31] simulated the fracture toughness tests of 304 stainless steel single edge bend specimens by using CZM in the absence and presence of hydrogen, respectively. Wu [32] used a two-dimensional (2D) FEM with cohesive elements to model the crack extensions in arc-shaped hydrogen-charged specimens of conventionally forged (CF) 21-6-9 austenitic stainless steel. The coupling effect of hydrogen diffusion in metal and the CZM based on hydrogen degradation law were not considered. In order to include the HEDE mechanism in the numerical analysis, many researchers made an attempt to obtain the HE phenomenon through quantum mechanics based on first principles to calculate the effect of hydrogen on decohesion [33,34,35]. Jiang and Carter [36] calculated that the ideal fracture energy nearly decreased in linearity with increasing hydrogen coverage based on periodic density functional theory. Serebrinsky et al. [37] presented a numerical mode for damage analysis in high-strength steel, accounting for the hydrogen influence in the HEDE mechanism, and the simulation results were in good agreement with the experimental results. Sobhaniaragh et al. [38] proposed fully coupled hydrogen transport and elastic-plastic deformation within CZM framework to predict hydrogen-induced cracking in high strength steels. The simulation of hydrogen-assisted fracture is a complex calculation process, including the transient hydrogen diffusion requiring the calculation of the strain-stress field of elastic-plastic material and the effect of hydrogen degradation on the promoted material fracture must be carried out into the CZM [39,40,41]. However, the macroscopic characteristic curve and microscopic fracture mechanism of hydrogen embrittlement can be obtained by in-depth analysis of the test process. It is also very time consuming to obtain full test characteristic data of a material using this methodology. Henceforth, it is necessary to study hydrogen embrittlement based on the combination of experimental investigations and finite element analyses (FEA) analysis.

The main objective of study was to provide a practical numerical tool to predict hydrogen-assisted brittle fracture response based on experimental results. First, CT specimens made of low alloy 30CrMo steels were hydrogen-charged, following which the fracture toughness test was performed, and then the failure microstructural characterization was carried out by scanning electron microscopy (SEM). Second, a 2-D plane strain FE was employed to simulate the hydrogen-assisted brittle fracture behavior by using ABAQUS software, which was approached for implementing the three steps including elastic-plastic stress analysis, hydrogen diffusion analysis and hydrogen-influenced cohesive zone stress analysis. A HEDE mechanism was adopted to simulate hydrogen-assisted fracture. The trapezoidal traction separation law (TSL) was devised, and the TSL parameters were calibrated by fitting the load-displacement and J-integral experimental curves in the absence of hydrogen. Furthermore, the influence of hydrogen concentration on the hydrogen coverage and hydrogen decreasing factor profile ahead of the crack tip were also evaluated. Finally, the numerical results for the hydrogen-charged specimens were compared with the experimental data.

## 2. Material and Experiment

**Figure 1:**
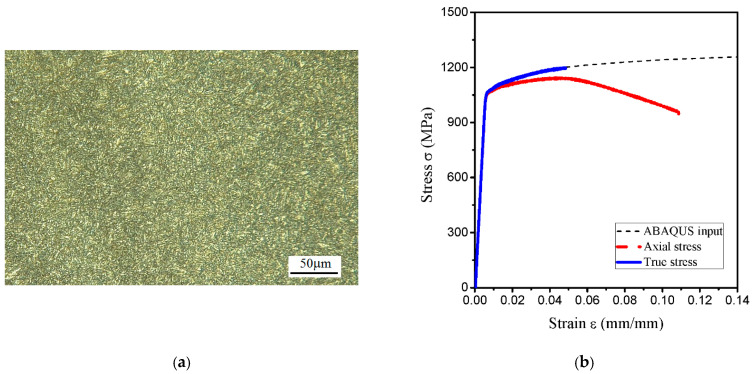
(**a**) Microstructure of the tested specimen and (**b**) experimental stress-strain curve obtained from the uniaxial tests.

In this study, a cylindrical bar of low alloy 30CrMo steel with a diameter of 60 mm was used as research material. The chemical compositions of low alloy 30CrMo are summarized in Table 1. The material was heated at 880 °C for 1 h, quenched into oil, tempered at 540 °C for 1 h and gradually cooled down to room temperature. Then the specimens were cut, ground and mechanically polished by grinding, which were then chemically corroded in a solution of 96% nitric acid and 4% alcohol to characterize the phase, structure, and grain boundaries of the compositions. The as-obtained microstructure shows the presence of tempered martensite, as presented in Figure 1a. The stress–strain curves were obtained by the uniaxial tests performed on the smooth round tensile bars (with a diameter of 6 mm) at room temperature, in accordance with ASTM E8 standard. The tests were carried out using an MTS809 axial-torsion servo hydraulic fatigue testing machine (MTS Systems Corporation, Eden Prairie, MN, USA). The longitudinal deformation was measured using an extensometer (MTS Systems Corporation, Eden Prairie, MN, USA) with a calibrated length of 25 mm. The mechanical properties of this steel are as follows: Young’s modulus 196 GPa, initial yield stress 1050 MPa, and Poisson’s ratio 0.3. The isotropic von Mises plasticity model was applied and the true stress relation to true plastic strain was defined by using the Hollomon hardening law: $\sigma_{t}=1370\cdot {\left(\varepsilon_{p}\right)}^{0.043}$, as shown in Figure 1b.

**Figure 2:**
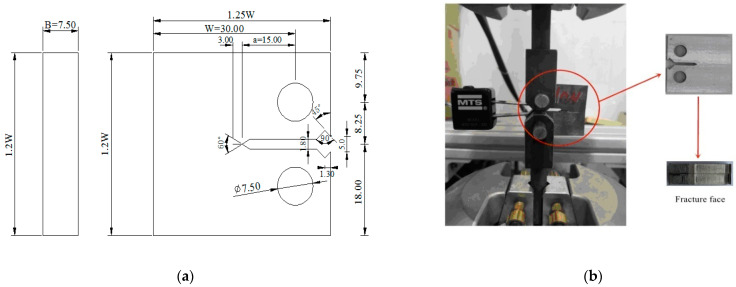
(**a**) Sketch and dimensions (mm) of compact tension (CT) specimen and (**b**) experiment process in CT specimen.

Fracture toughness tests were determined using CT specimens with a notch length of 15 mm, a width of 30 mm, and a thickness of 7.5 mm, as shown in Figure 2a. The orientations of CT specimens in this investigation are designed to be along the radial direction of the round bar. The specimens were first fatigue pre-cracked before the experiment. Fatigue tests on pre-cracked specimens were carried out on an MTS809 axial-torsion servo hydraulic fatigue testing machine (MTS Systems Corporation, Eden Prairie, MN, USA) under a frequency of 5 Hz and a load ratio R=0.1 until an initial crack length versus width ratio of a/W = 0.6 was acquired, following the ASTM E1820 standard [42]. CT specimens were charged in 0.1 mol/L NaOH solution at a constant current density of 2 mA cm^−^^2^ by the electrochemical method. The specimen was employed as the cathode and a carbon rod was used as the anode. The definition of the maximum hydrogen diffusion distance (x) as a function of the hydrogen diffusion coefficient (D) and hydrogen charging time (t) is expressed as follows [43]:

(1) $$x=4\sqrt{Dt}$$

The value of hydrogen diffusion coefficient (D) was selected according to the literature study [32] to be about 10^−5^ mm^2^ s^−^^1^ for low alloy martensitic steels. By substituting x = 3.75 mm and D = 10^−5^ mm^2^ s^−1^ into Equation (1), the calculated hydrogen charging time was about 24 h. In order to study the effect of different initial hydrogen content on the mechanical properties of the steel, hydrogen charging was carried out for 12, 24 and 36 h in the present study. To prevent the amount of hydrogen escaping from hydrogen-charged specimens, specimens were carried out immediately after hydrogen charging.

Hydrogen-charged specimens were investigated in air under a lower nominal displacement rate of 0.002 mm s^−^^1^ in order to study the influence of HE with the longer diffusion time. Moreover, an uncharged specimen was also tested as a reference. The MTS 632.02F-20 COD extensometer (MTS Systems Corporation, Eden Prairie, MN, USA) was used to measure the crack mouth opening displacement during the experiment. The mechanical experimental process is represented in Figure 2b. Measurement of the crack length, a, was obtained by compliance method based on the following equations [44]:

(2) $$\frac{a}{W}=1.0010-4.6695u_{x}+18.460u_{x}^{2}-236.82u_{x}^{3}+1214.9u_{x}^{4}-2143.6u_{x}^{5}$$

(3) $$u_{x}={\left({\left[\frac{EV_{0}B}{P}\right]}^{1/2}+1\right)}^{-1}$$

where a is the crack length, W is the specimen width, E is the Young’s modulus, $V_{0}$ is the crack mouth opening displacement, and P is the applied load.

The calculation of load-line displacement ($V_{ll}$) was based on the linear elastic relationship with the crack mouth opening displacement, which is represented as follows [45]:

(4) $$\{\begin{array}{c} V_{ll}/V_{0}=f\left(\frac{a}{W}\right)\\ f\left(\frac{a}{W}\right)=0.2255+1.8352\left(\frac{a}{W}\right)-2.8046{\left(\frac{a}{W}\right)}^{2}+1.8742{\left(\frac{a}{W}\right)}^{3}+0.3276{\left(\frac{a}{W}\right)}^{4}-0.6812{\left(\frac{a}{W}\right)}^{5} \end{array}$$

**Figure 3:**
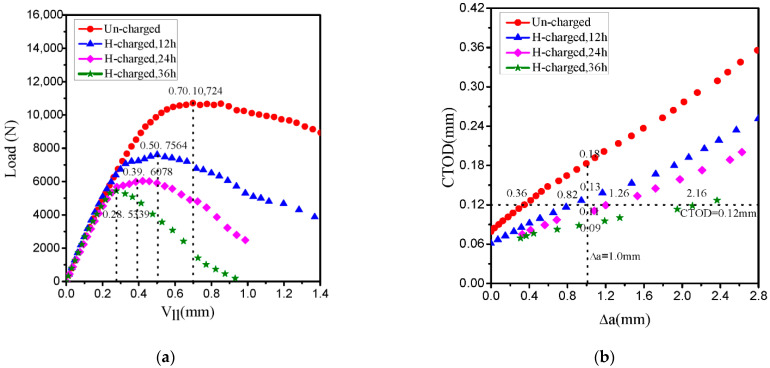
(**a**) Load-line displacement and (**b**) crack growth resistance (CTOD-R) curves for uncharged and hydrogen-charged specimen.

The load-line displacement ($V_{ll}$) curves of the uncharged and hydrogen-charged specimens for 12, 24 and 36 h, are plotted in Figure 3a. The maximum load shows a distinct trend for the hydrogen-charged specimens. The black dotted vertical lines indicate the maximum load and the corresponding load-line displacement on the curves. Maximum HE occurred under hydrogen-charged time of 36 h, and the effect of hydrogen on mechanical properties was obvious: maximum load and corresponding load-line displacement decreased by 50% and 60%, respectively. This is attributed to the fact that when the specimen was charged with hydrogen for 36 h, which exceeded the theoretical calculation for 24 h, hydrogen atoms could transport into the center regions, leading to the deterioration of mechanical properties of 30CrMo steel. The crack growth resistance (CTOD-R) curves of the uncharged and hydrogen-charged specimens for 12, 24 and 36 h, are shown in Figure 3b. Analysis of graphs revealed that the lower curves for hydrogen-charged specimens indicate that the steel undergoes a significant degradation due to dissolved hydrogen, giving rise to the higher crack propagation and the lower crack growth resistance, and similar curve trend has been found in previous study by Scheider et al. [46]. However, the CTOD-R curves show a decreasing trend in fracture toughness with the increase in the hydrogen charging time. Indeed, taking the fixed value of crack extension as, Δa = 1.0 mm, the black dotted vertical lines corresponding to the figure, the crack tip opening displacement values reduce by about 1/3 from uncharged to hydrogen-charged for 12 h (the corresponding figure data are 0.18 to 0.13 mm), and by 1/6 from 24 to 36 h (the corresponding figure data are 0.11 to 0.09 mm). For the same fixed value of crack tip opening displacement, CTOD = 0.12 mm, the black dotted horizontal lines corresponding to the figure, the crack extension values increase by 0.46 mm from uncharged to hydrogen-charged for 12 h (the corresponding figure data are 0.36 to 0.82 mm), and by 0.9 mm from 24 to 36 h (the corresponding data are 1.26 to 2.16 mm). It is well known that hydrogen atoms diffuse and accumulate ahead of the crack tip (driven by the high hydrostatic stress existing at the crack front) when a mechanical load is applied to the hydrogen-charged specimen, giving rise to the HE phenomenon.

**Figure 4:**
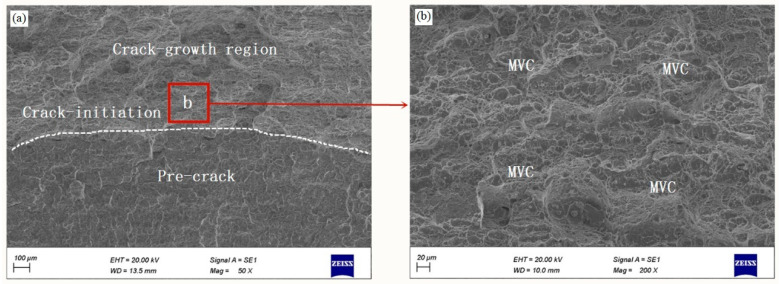
(**a**) Scanning electron microscopy (SEM) images of the fracture surface for uncharged specimen, and (**b**) SEM high magnification image of the indicated region in (**a**).

The fracture surfaces of broken specimens were observed by SEM (ZEISS Gemini SEM500, CarlZeiss, Oberkochen, Germany) under an acceleration voltage of 20 kV, and only the fracture surfaces of CT specimens at the appropriate locations were illustrated. The fracture surface of uncharged specimen is displayed in Figure 4a. The fracture surfaces consist of three regions, i.e., pre-crack region, crack initiation region and crack growth region. Figure 4b presents a higher-resolution SEM image of the central crack initiation region in Figure 4a. The entire fracture surface exhibits ductile fracture mode with a range of deep dimples fracture mode, as illustrated by fully ductile MVC.

**Figure 5:**
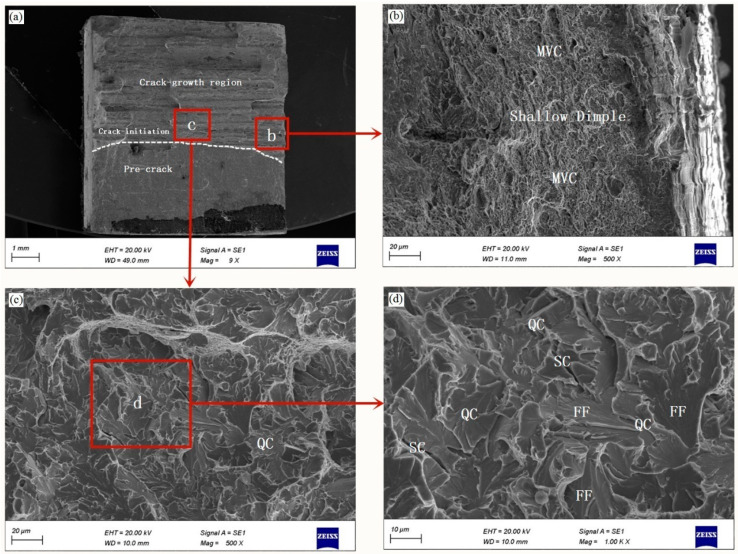
SEM images of the fracture surface of specimen charged for 12 h, and (**a**) overview of the fracture surface; (**b**,**c**) High magnification SEM images of the indicated region in image (**a**); (**d**) High magnification SEM image of the indicated region in image (**c**); QC: quasi-cleavage; MVC: micro-void coalescence; FF: flat facet, SC: secondary crack.

Figure 5 presents the fracture surfaces associated with specimens charged with hydrogen for 12 h. Figure 5a shows a general view of the fractured surface, where two different fracture regions can be distinguished: pre-crack region, with plane facets and ductile striations (similar features regardless whether the specimens were charged with hydrogen or not (see Figure 4a)), while fracture region consists of the crack initiation region and the crack growth region. Figure 5b exhibits the enlarged view of the edge of fracture surfaces of a small region shown in the SEM image in Figure 5a. The fracture mode is characterized by a mixed fracture micromechanism composed of plasticity-related hydrogen induced fracture in a general MVC micromechanism. Compared to the uncharged specimen, the fracture exhibits fracture mode with smaller and shallower dimples. Figure 5c presents the magnified view of a small central crack initiation region in Figure 5a. The fracture surfaces exhibit QC fracture with flat facets (FFs) and river markings features, which correspond to typical features of hydrogen-assisted fracture [47]. A high-magnification image of the QC fracture is given in Figure 5d, which clearly shows such fine, lath-like features accompanied by fine serrated markings and deep secondary cracks (SCs), which is shown as the typical brittle fracture mode. It is thus confirmed that the fracture surfaces of hydrogen-charged specimen for 12 h shows a mixture of QC fracture and ductile MVC features.

**Figure 6:**
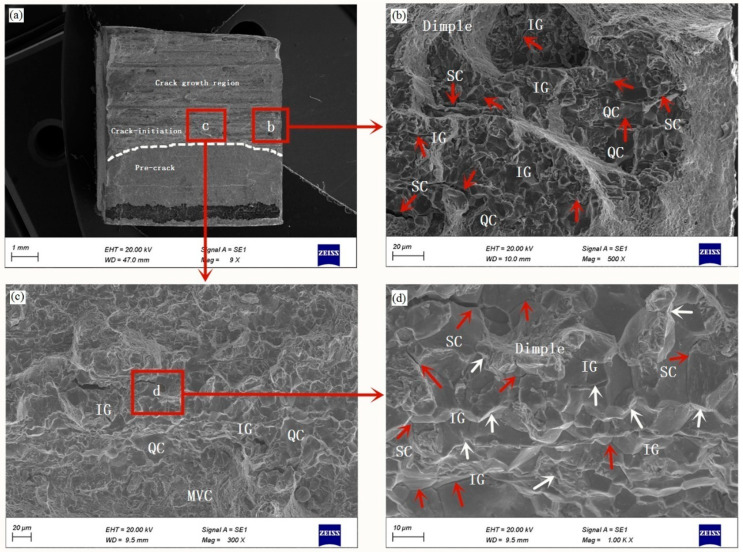
SEM images of the fracture surface of specimen charged for 36 h, and (**a**) overview of the fracture surface; (**b**,**c**) High magnification SEM images of the indicated region in image (**a**); (**d**) High magnification SEM image of the indicated region in image (**c**); (Red arrowheads indicate secondary cracks (SC) and white arrowheads indicate tear ridges). QC: quasi-cleavage; IG: intergranular fracture; MVC: microvoid coalescence.

The fracture surfaces of specimens after charging for 36 h are shown in Figure 6. The general view of the fractured surface exhibits similar features as those observed for the specimens charged for 12 h (see Figure 5a). The high-magnification images of the central crack initiation region and the edge of the fracture region are shown in Figure 6b,c, respectively. The fracture surfaces show a mixed brittle and ductile mode of fracture, such as QC, IG fracture and some evidence of small-scale MVC fracture occupying other areas. The high-magnification SEM image (Figure 6d) indicates that the fine tear ridges (marked with white arrowheads) and intergranular secondary cracks (marked with red arrowheads) are observed on the fracture surface. Hydrogen-assisted fracture mode depends on mechanical properties, hydrogen concentration and microstructure [48]. The microscopic mechanism can be explained as follows: hydrogen atoms diffuse and accumulate ahead of the crack tip, where the maximum hydrostatic stress is located in this region [49,50]. When the hydrogen concentration of GB exceeds a critical value, the cohesive strength of GB is weakened and the GB separations occur, leading to the HEDE failure mechanism. The HEDE failure mechanism appears to occur and gives rise to IG fracture. The IG fracture and QC fracture modes have been observed and studied for hydrogen-charged tempered high strength low-alloy steels [51,52]. In summary, the fracture surface morphology of hydrogenated specimens are followed by the change from the QC and MVC mode with reduced dimple sizes due to HELP mechanism dominance at lower content of diffused hydrogen. Then, the transition to predominately brittle mixed QC and IG fracture mode occurs due to the HEDE mechanism activation and exceeding the critical hydrogen concentration for their separations. Recently, Kumar et al. [53] reported that HEDE mechanism was the dominant mechanism in tempered 13 wt % Cr martensitic stainless steel when apparent hydrogen concentration was higher. The present study carried out the experiments under serious hydrogen charging conditions and could obtain high hydrogen concentration in specimens; however, the 30CrMo steel specimens exhibited higher HE susceptibility and IG fracture mode after hydrogen-charging for 36 h, in which the failure mechanism followed the HEDE mechanism.

## 3. Model Framework

The aim of the model is to provide a numerical tool to calculate and predict hydrogen-assisted fracture behavior. The model geometry represents a two-dimensional discretization of the CT specimen used for the fracture mechanics testing. The HE simulation procedure (ABAQUS 6.14, (dassault)SIMULIA, Providence, RI, USA) needs to be implemented in the three steps following the work of Olden et al. [34,54], which include elastic-plastic stress analysis, hydrogen diffusion analysis, and the final elastic-plastic analysis with addition of user-defined cohesive elements in the crack ligament area. The coupled problem of transient diffusion-mechanics was solved in a simulation procedure. In the first step, the stress field was given as input for the following hydrogen diffusion analysis. In the second step, the hydrogen diffusion ahead of the crack tip analysis was based on the stress field previously calculated. In this last step, the hydrogen concentration computed in the previous step was imported and hydrogen-influenced cohesive stress was implemented by altering a TSL. Further details of the simulation process are described as follow.

### 3.1. The First Step: Elastic-Plastic Analysis

The standard von Mises plasticity model was used for the elastic-plastic stress analysis, and the elastic-plastic parameters are obtained by the previous uniaxial test. The model is loaded to a certain load level and generates the stress field information under the control of displacement. The stress field information conducts outputs in the result Abaqus file to provide an input for hydrogen diffusion analysis.

### 3.2. The Second Step: Hydrogen Diffusion Analysis

Hydrogen atoms adhere to different positions of the metal, i.e., in lattice sites $C_{L}$ and trapping sites $C_{T}$ at a variety of defect microstructures such as dislocations, GB, and carbides. The stress field influences hydrogen diffusion by means of two phenomena: hydrostatic stress, which produces lattice dilatation so hydrogen will tend to reach expanded sites, and plastic strain, which increases the amount of crystal defects creating trapping sites.

Stress-driven hydrogen diffusion analysis is defined as an extension of Fick’s law The driving force for diffusion is the chemical potential gradient ∇u, The mass flux is related to ∇u via Onsager coefficients $L_{ij}$, which denote the action of force *j* on component *i*; a negative sign indicates that the net movement of *i*-type hydrogen atoms, i.e., hydrogen flux $J_{i}$, occurs from high to low chemical potential regions:

(5) $$J_{i}=-\sum_{j=1}^{n}L_{ij}\nabla u_{j}$$

In particular, for lattice and trapping sites, fluxes might be expressed in a matrix form:

(6) $$\left[\begin{array}{c} J_{L}\\ J_{T} \end{array}\right]=\left[\begin{array}{cc} L_{LL} & L_{LT}\\ L_{TL} & L_{TT} \end{array}\right]\left[\begin{array}{c} \nabla u_{L}\\ \nabla u_{T} \end{array}\right]$$

However, it is usually assumed that the lattice chemical potential $u_{L}$ does not affect the flux between trapping sites and the trapping chemical potential $u_{T}$ does not affect the flux between lattice sites. Cross-terms are thus neglected, $L_{LT}=L_{TL}=0$, so:

(7) $$J_{L}=-L_{LL}\nabla u_{T}$$

(8) $$J_{T}=-L_{TT}\nabla u_{T}$$

The Onsager coefficient is related to Einstein’s equation of diffusion:

(9) $$L_{LL}=\frac{D_{L}}{R\left(T-T_{z}\right)}C_{L}$$

(10) $$L_{TT}=\frac{D_{T}}{R\left(T-T_{z}\right)}C_{T}$$

where $D_{L}$ is the lattice diffusivity, $D_{T}$ is the diffusivity between trapping sites, T is the temperature, $T_{Z}$ is the absolute temperature, and R is the universal gas constant.

Here, assuming that the mobility between trapping sites is considered close to zero: $D_{T}=0$, because traps are not connected or because their deep potential energy prevents hydrogen from diffusing well. The driving force for diffusion is the chemical potential gradient, and fluxes might be expressed as follows:

(11) $$J_{L}=-\frac{D_{L}C_{L}}{R\left(T-T_{z}\right)}\cdot \nabla \mu_{L}$$

(12) $$J_{T}=0$$

The lattice chemical potential $\mu_{L}$ is defined as follows:

(13) $$\mu_{L}=\mu_{0}+R\left(T-T_{z}\right)\ln\phi +pV_{H}$$

where $\mu_{0}$ is the chemical potential at standard condition, $V_{H}$ is the partial molar volume of hydrogen, ϕ is the normalized hydrogen concentration. The normalized hydrogen concentration is defined as $\phi =C_{L}/s$ denoting the relation between the mass concentration of the diffusing material $C_{L}$, $s=4300\cdot e^{-3261/\left(T-T_{z}\right)}$ is the solubility of hydrogen. By substituting Equation (13) into Equation (11) we obtain:

(14) $$J_{L}=-sD_{L}\cdot \left[\frac{\partial \phi }{\partial x}+k_{s}\frac{\partial }{\partial x}\left[\ln\left(T-T_{z}\right)\right]+k_{p}\frac{\partial p}{\partial x}\right]$$

where D is the diffusion coefficient for hydrogen. $k_{s}=\phi \ln\phi$ is the “Soret effect” factor, providing diffusion because of temperature gradient and $k_{p}=\frac{V_{H}\phi }{R\left(T-T_{z}\right)}$ is the pressure stress factor, providing diffusion driven by the gradient of the equivalent pressure stress, $p=-trace\left(\sigma \right)/3=-\sigma_{h}$, $\sigma_{h}$ is hydrostatic stress.

The mass conservation equation is expressed as follows:

(15) $$\frac{\partial C_{L}}{\partial t}=-\nabla \cdot J_{L}$$

The experimental test was carried out immediately after hydrogen charging. The temperature variation was very small during the test. Taking no account of the temperature gradient and substituting Equation (14) into Equation (15), the diffusion equation under the gradient of stress is obtained [32]:

(16) $$\frac{\partial C_{L}}{\partial t}=D_{L}\nabla^{2}C_{L}+D_{L}\cdot \frac{V_{H}}{R\left(T-T_{z}\right)}\nabla C_{L}\cdot \nabla p+D_{L}\cdot \frac{V_{H}}{R\left(T-T_{z}\right)}C_{L}\cdot \nabla^{2}p$$

Taha and Sofronis [27] revealed the characteristic distributions of hydrogen in trapping sites and the plastic strain exhibited a similar trend around the crack tip. Actually, if we assume that the content of hydrogen in trapping sites are always in equilibrium with those in lattice sites. Both of these contribute to hydrogen-induced fracture and need to be taken into account. The relation used in this work, a linear relationship between the hydrogen in trapping sites and the plastic strain, was proposed by Olden et al. [34]:

(17) $$C_{T}=(49\cdot \varepsilon_{p}+0.1)\cdot C_{L}$$

The lattice hydrogen concentration results were calculated from Equation (16), and the trapping hydrogen is considered to be caused by plastic strain near the crack tip after applying stress, which can be obtained from Equation (17). In this paper, the author considers only one kind of trap, i.e., hydrogen trapped at dislocations. The total concentration of hydrogen is given by $C=C_{L}+C_{T}$.

### 3.3. The Third Step: Cohesive Analysis

The CZM was first posed by Dugdale [55] and Barenblatt [56], who used to simulate the material separation in the process of fracture by defining a suitable TSL. For the present study, a trapezoidal TSL was used, which was proposed by Scheider et al. [46]. The trapezoidal traction separation law consists of four polynomials, defined as follows:

(18) $$T\left(\delta \right)=\{\begin{array}{cc} \frac{\sigma_{\max}}{\delta_{1}}\cdot \delta & \delta <\delta_{1}\\ \sigma_{\max} & \delta_{1}\leq \delta \leq \delta_{2}\\ \frac{\sigma_{\max}}{\delta_{f}-\delta_{2}}\cdot \left(\delta_{f}-\delta \right) & \delta_{2}\leq \delta \leq \delta_{f}\\ 0 & \delta >\delta_{f} \end{array}$$

where T(δ) is the traction, $\sigma_{\max}$ is the critical cohesive strength, and $\delta_{f}$ is the critical separation. The $\delta_{1}$ and $\delta_{2}$ are the critical separation in the linear part and the plastic stage of the TSL, respectively.

The area inside TSL curve represents the cohesive energy $T_{c}$, the equation is defined by:

(19) $$T_{c}=\frac{1}{2}\sigma_{\max}\left(\delta_{f}+\delta_{2}-\delta_{1}\right)$$

**Figure 7:**
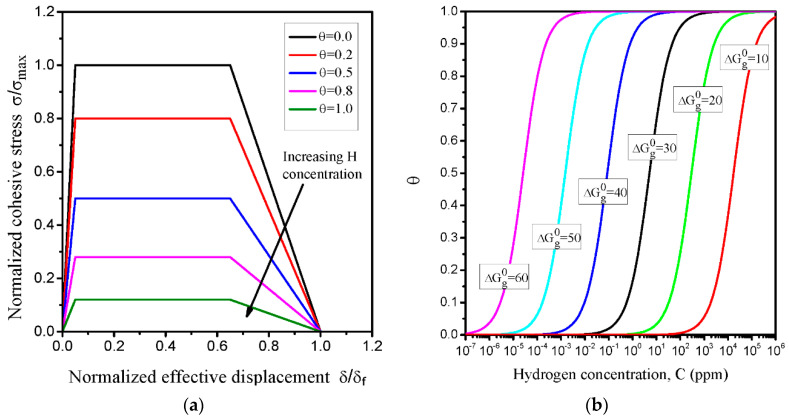
(**a**) Trapezoidal traction-separation law (TSL) for various values of hydrogen coverage θ; (**b**) hydrogen coverage with hydrogen concentration for various levels of Gibbs free energy range of 10 to 60 kJ·mol^−1^.

The reduction of cohesive energy calculated with increasing hydrogen coverage θ was originally proposed by Serebrinsky et al. [37], the calculation of breaking energy based on experimental data was reported by Jiang and Carter [36], and the current cohesive strength $\sigma {\left(\theta \right)}_{\max}$ associated with the local critical hydrogen concentration is expressed as follows:

(20) $$\frac{\sigma {\left(\theta \right)}_{\max}}{\sigma {\left(0\right)}_{\max}}=1-1.0467\theta +0.1687\theta^{2}$$

where θ is the hydrogen coverage, $\sigma {\left(0\right)}_{\max}$ is the cohesive strength with no hydrogen influence and $f(\theta )=1-1.0467\theta +0.1687\theta^{2}$ is the decreasing factor. Figure 7a shows the effect of the coverage factor θ on the normalized trapezoidal TSL curve.

Relationship between the hydrogen concentration C and hydrogen coverage θ, from the Langmuir–McLean isotherm is defined as follows [36,57]:

(21) $$\theta =\frac{C}{C+\exp(-\Delta G_{g}^{0}/RT)}$$

where $C=C_{L}+C_{T}$ is the sum of trapped and lattice hydrogen populations, $\Delta G_{g}^{0}$ is the Gibbs free energy, T is the temperature and R is the universal gas constant. The curves of hydrogen coverage with hydrogen concentration for various levels of Gibbs free energy range of 10 to 60 kJ mol^−^^1^ are shown in Figure 7b. Clearly, hydrogen concentration covers about four orders of magnitude for a given Gibbs free energy. The lower boundary and the upper boundary represent the hydrogen concentration at fracture initiation and the ultimate saturation level, respectively. Therefore, the value of Gibbs free energy is very important and cannot be determined arbitrarily. Following the study by Serebrinsky et al. [37], the Gibbs free energy $\Delta G_{g}^{0}$ is assigned a value of 30 kJ mol^−^^1^, representing the initiation hydrogen concentration and an ultimate saturation level ranging between 0.01 and 100 ppm in the α-Fe grain boundary.

## 4. Finite Element (FE) Computational Model

**Figure 8:**
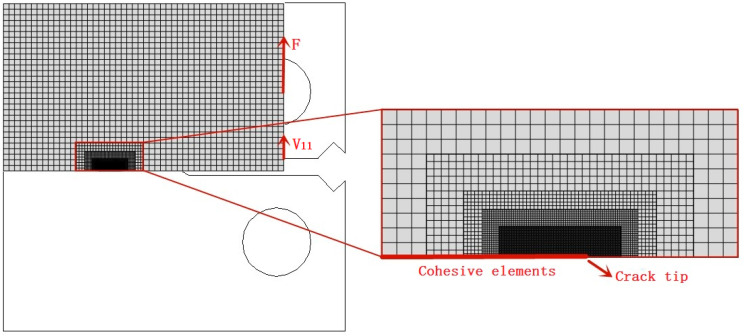
The finite element (FE) mesh (without the hole) structure around the initial crack tip.

A 2D finite element model (ABAQUS 6.14 (dassault)SIMULIA, Providence, RI, USA) was established to study experimental testing. Considering the geometric symmetry of the model, only the upper half of the specimen was modeled, as shown in Figure 8. Boundary conditions were related to geometric symmetry and the vertical load F was applied directly to a vertical strip of nodes in the hole region. During the elastic-plastic analysis, the four-node plane strain elements (CPE4R) were adopted for the continuum elements. The number of continuum elements is 7 765. The experimental true stress-plastic strain data for material were obtained by laboratory tensile test, reported in Table 2. In order to ensure sufficient resolution of the local stress and strain after loading, the mesh was locally refined at the crack tip and along the ligament of the specimen with the smallest element length of 18.75 μm. A mesh sensitivity study was considered by using two different finite element meshes with the smallest sizes of 62.50 μm and 18.75 μm. The computational results showed that the difference in stress field information near the crack tip was very small. In the mass diffusion analysis, the concentration field of hydrogen uses 2D elements (DC2D4). In the cohesive analysis, the cohesive zone was predefined and uniformly distributed using zero thickness cohesive elements (COH2D4) along the crack growth path. The number of cohesive elements was 133, and the element sizes range from 18.75 μm to 0.6 mm. Li et al. [58] reported that the cohesive model was not sensitive to the element size in a large number of crack fracture calculations. In order to implement the three-step calculation process, the same nodes and elements were used in the three steps. Finally, data collection point is set on the model loading line to simulate load-line displacement V_ll_. Table 3 lists the values of the parameters adopted in the model.

### 4.1. Selection of Trapezoidal Traction-Separation Law (TSL) Parameters

**Figure 9:**
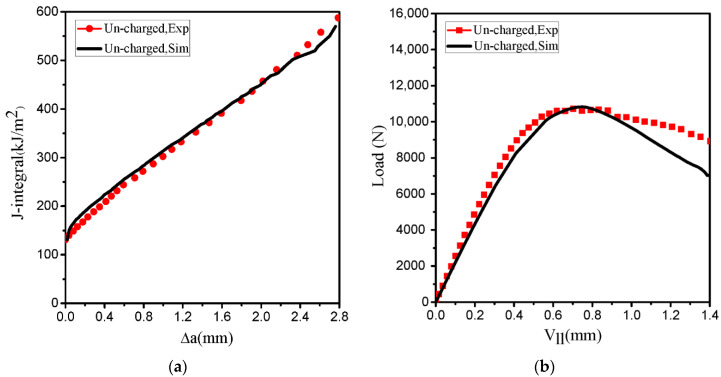
(**a**) The J-R curves of the uncharged specimen and (**b**) load-displacement curves of uncharged specimen corresponding to computational and experimental data. (Exp: experiment; Sim: simulation).

The cohesive simulation assumes that the model fails under the condition of crack initiation, and the complete separation of the first cohesive element is considered as a complete failure. In the present study, the cohesive elements start to damage when the maximum cohesive strength reaches the critical cohesive strength $\sigma_{\max}$, and the crack tip is defined at the location where the critical separation $\delta_{f}$ just reaches. Therefore, the appropriate selection of TSL parameters is very important. The cohesive energy $T_{c}$ is represented by the area below the trapezoidal TSL curve, which determines the fracture behavior. The unit of J-integral and cohesive energy $T_{c}$ are the same as per unit area below the TSL. The cohesive energy $T_{c}$ is obtained from the combination of the cohesive strength $\sigma_{\max}$ and the critical separation $\delta_{f}$. In this investigation, the ratio $\delta_{1}/\delta_{f}$ and $\delta_{2}/\delta_{f}$ are selected to be fixed values of 0.05 and 0.65 for all simulations, respectively [40]. Therefore, the cohesive energy $T_{c}$ and the critical cohesive strength $\sigma_{\max}$ are selected as two independent parameters to be applied to the simulation. Based on previous experiments, the experimental results of the J-integral curve are presented in Figure 9a. In this study, the cohesive energy is selected based on the J-integral of the uncharged specimen at crack initiation from the experiment [31]. The cohesive energy $T_{c}$ at the crack initiation is selected to be $132\;\mathrm{KJ}/m^{2}$ for an uncharged specimen (Figure 9a). The upper half of the FE is used for simulation; therefore, the cohesive energy $T_{c}$ is half of the initiation cohesive energy of the experiment. The numerical calculation results from the selection of the cohesive energy corresponding to the J-integral at crack initiation indicate that the J-R and the load-displacement ($V_{ll}$) curves are in accordance with the experimental data (Figure 9a,b). Therefore, the independent TSL parameters providing the best fit to experimental data for the trapezoidal TSL are selected as the cohesive strength of 2100 MPa, which equals $2\sigma_{y}$, and 0.0393 mm for the critical cohesive separation $\delta_{f}$. Table 4 lists the TSL parameters for the uncharged specimen.

### 4.2. Implementing Hydrogen-Influenced TSL

This section mainly describes the approaches for implementing the coupling relationship between hydrogen diffusion and CZM. In the step of hydrogen diffusion analysis, a uniform hydrogen distribution through the specimen $C(t=0)=C_{L0}$ (as is the case in experiments, where the specimen is pre-charged) is prescribed as an initial condition. The outer surfaces of specimens are assumed to have zero hydrogen flux boundary conditions, which are prescribed as $C_{b}=C_{L0}$. The initial hydrogen concentration is defined as a predefined field as follows: *Initial Condition, type = CONCENTRATION. The hydrogen diffusion analysis shows the normalized concentration ϕ, NNC. In the cohesive analysis, the normalized hydrogen concentration ϕ, NNC, is read from the database of the previous step and the following step is imported as the value of predefined field (FV1), which is defined as follows: *Initial Conditions, type = FIELD, variable = 1, file = file-name, output variable = NNC, interpolate. It is noteworthy that in order to calculate the trapping sites $C_{T}$, the UVARM subroutine was used. The UVARM calls the utility routine GETVRM to provide access to material integration point data for each increment of the step. The equivalent plastic strain $\varepsilon_{p}$ is obtained by the utility routine GETVRM. Then, the hydrogen in lattice sites $C_{L}$ is calculated by Equation (16), $C_{T}$ can be obtained from Equation (17) and the total concentration can be calculated by *C* = *C_L_* + *C_T_*, with θ taken from Equation (21) and f(θ) taken from Equation (20). The f(θ) factor is defined as field variable with the USDFLD subroutine and is stored in a common block to transfer from the continuum elements to the adjoining cohesive elements. Finally, the effect of hydrogen degradation on the promoted material fracture is implemented into the TSL.

## 5. Numerical Results and Discussion

**Figure 10:**
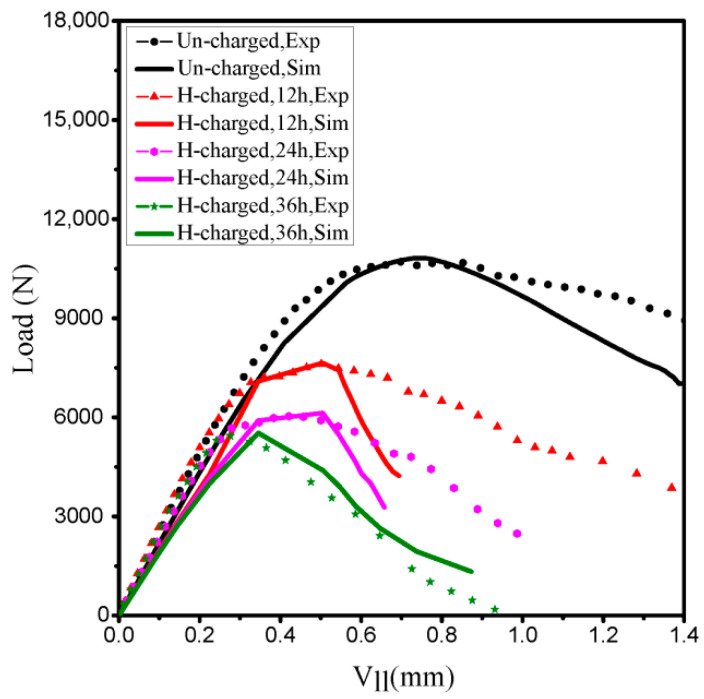
$F-V_{\mathrm{ll}}$ curves from experimental data and results of numerical simulations. (Exp: experiment; Sim: simulation).

Electrochemical pre-charged hydrogen technique shows that hydrogen has an obvious effect on the mechanical properties of the steel; nonetheless, the limitation of this technology lies in the lack of effective methods to accurately measure the hydrogen distribution and content in steel. According to the HEDE mechanism and FEM, the effective initial hydrogen content can be estimated by using a numerical model. Using the above three steps in finite element (FE) model analysis, the initial hydrogen concentration $C_{L0}$ defines a predefined field (FV1), and the elastic-plastic and TSL parameters remain constant for all simulations. After several iterations of simulation, the load-line displacement ($V_{ll}$) curves obtained from numerical analysis provide the best fit to the experimental curves. The variation of the applied load with load–line displacement for different initial hydrogen concentrations is shown in Figure 10. The general trends of the simulation and the experiment curves are consistent. The normalized hydrogen concentration is defined as $\phi =C_{L}/s$, and the solubility of hydrogen s is 0.071 ppm mmN^−1/2^. As a result, three different initial normalized concentrations corresponding to hydrogen-charged specimens for 12, 24 and 36 h are 49.30, 77.46 and 119.72 NNC (corresponding to initial hydrogen concentration in lattice sites $C_{L0}$ of 49.30 × 0.071 = 3.5, 77.46 × 0.071 = 5.5 and 119.72 × 0.071 = 8.5 ppm), respectively.

**Figure 11:**
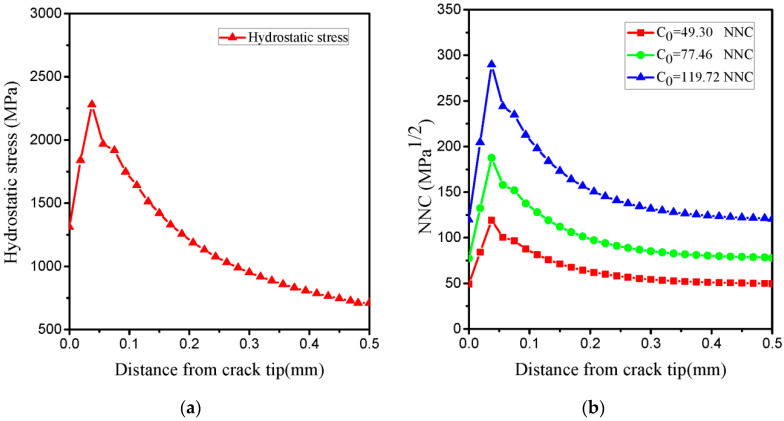
(**a**) Calculated hydrostatic stress distribution as a function of the distance from the crack tip; (**b**) calculated normalized hydrogen concentration NNC distribution as a function of the distance from the crack tip for $C_{L0}$ (49.30, 77.46 and 119.72 NNC). NNC: normalized hydrogen concentration.

**Figure 12:**
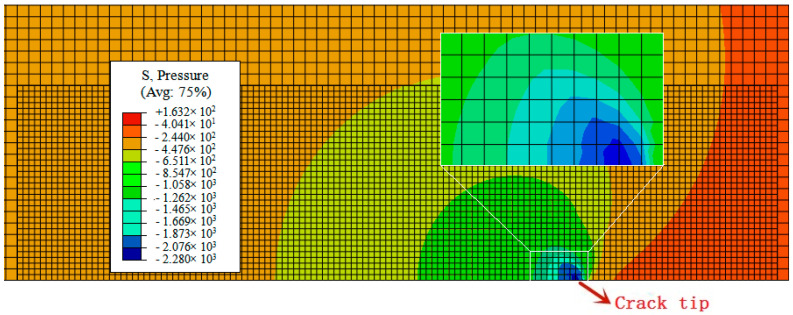
The contour plots of p hydrostatic pressure after the elastic-plastic analysis. Avg.: averaging threshold.

**Figure 13:**
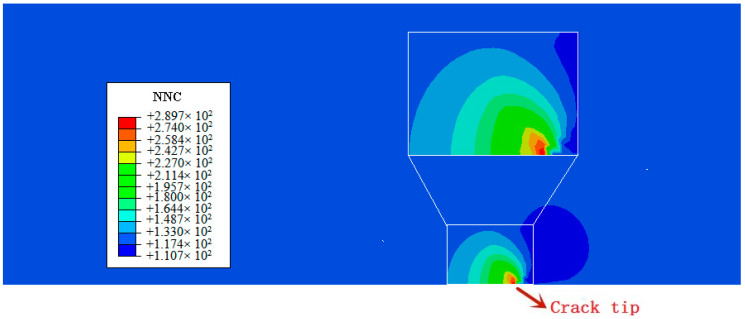
The contour plots of normalized hydrogen concentration after 600 s diffusion. NNC: normalized hydrogen concentration.

The calculated distribution of hydrostatic stress at the end of load for the elastic-plastic analysis as a function of the distance from the crack tip, is shown in Figure 11a. Figure 12 shows the contour plots of hydrostatic pressure after the elastic-plastic analysis. The results show that the maximum hydrostatic stress appears ahead of the crack tip. Figure 11b shows the calculated distribution of three normalized hydrogen concentrations at the end of diffusion time. Figure 13 exhibits the contour plots of normalized hydrogen concentration after 600 s diffusion for $C_{L0}$ (119.72 NNC, corresponding to initial hydrogen concentration of 8.5 ppm). Herein, in the simulation, the normalized hydrogen concentration is clearly observed near the first element ahead of the crack tip, and its peak corresponds with the peak of hydrostatic stress. The peak of hydrogen concentration is almost 2.4 times of the initial value, equal to 289.7 NNC at the end of diffusion time. Comparison of Figure 11a,b with Figure 12 and Figure 13 clearly indicates that the distribution of normalized hydrogen concentration is consistent with the hydrostatic stress. This result can be understood from the mass diffusion equation (recall Equation (16)), which causes hydrogen concentration to accumulate in the high hydrostatic stress region.

**Figure 14:**
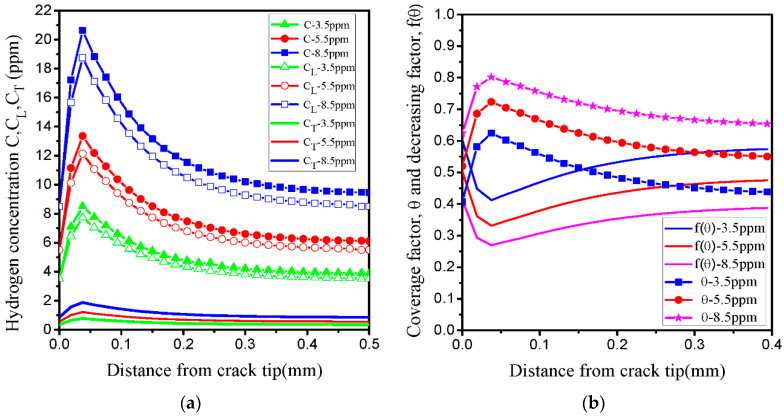
(**a**) Calculation of the hydrogen concentration ($C_{L}$, $C_{T}$, $C=C_{L}+C_{T}$) as a function of distance from the crack tip for $C_{L0}$ (3.5, 5.5, 8.5 ppm); (**b**) hydrogen coverage factor θ and hydrogen decreasing factor f(θ) as a function of distance from the crack tip for $C_{L0}$ (3.5, 5.5, 8.5 ppm).

**Figure 15:**
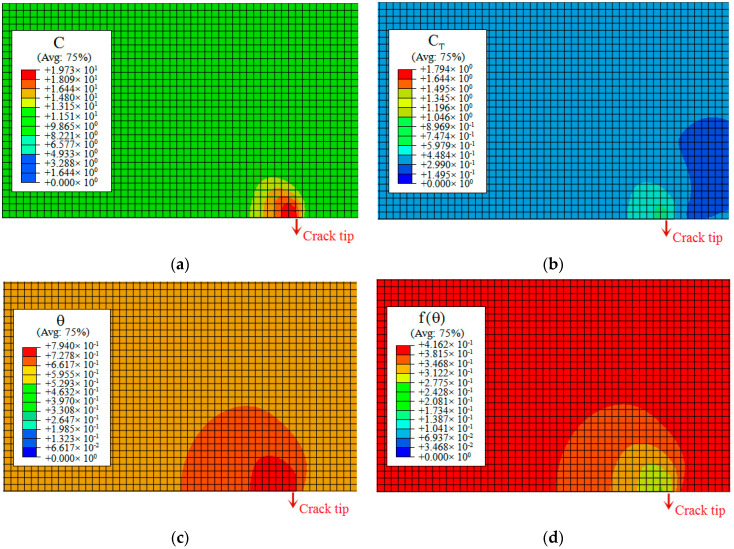
Results of simulations at the crack tip region for $C_{L0}$ = 8.5 ppm: (**a**) total hydrogen concentration $C=C_{L}+C_{T}$; (**b**) trapped hydrogen concentration $C_{T}$; (**c**) coverage factor θ trend; and (**d**) TSL decreasing factor f(θ) trend. Avg.: averaging threshold.

**Figure 16:**
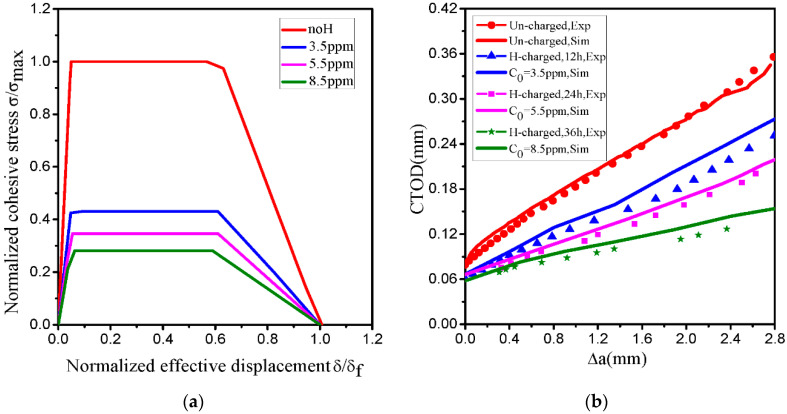
(**a**) TSL at various $C_{L0}$ (0.0, 3.5, 5.5, and 8.5 ppm); (**b**) CTOD-R curves from experimental data and results of numerical simulations. (Exp: experiment; Sim: simulation).

Figure 14a shows the distributions of the hydrogen concentration ($C_{L}$,$C_{T}$,$C=C_{L}+C_{T}$) as a function of the distance from the crack tip. The results show that the three types of hydrogen concentration are observed ahead of the crack tip; however, this decreases sharply at a distance from the crack tip. According to previous experimental results presented in Figure 1b, the equivalent plastic strain level is below 0.2. In our simulation, the hydrogen in trapping sites is not sufficient to significantly increase the total concentration ahead of the crack tip. This indicates that the hydrogen in lattice sites $C_{L0}$ plays a significant role in determining HE. Based on the HEDE theory [34,35,36,37], the cohesive energy is expressed as the area inside the TSL curve, which reduces with the change of local hydrogen concentration. Then, corresponding to the peak of the total hydrogen concentration $C=C_{L}+C_{T}$, the decreasing factor f(θ) reaches the minimum value ahead of the crack tip from Equation (20). Owing to the consideration of the decreasing factor f(θ), it is possible to simulate and predict hydrogen-assisted fracture. Figure 14b shows hydrogen coverage θ and decreasing factor f(θ) as a function of the distance from the crack tip. The contours of the four simulation results ($C_{T}$,$C=C_{L}+C_{T}$,θ,f(θ)) for the initial hydrogen concentration of 8.5 ppm are illustrated in Figure 15a–d, and the values of hydrogen coverage θ and decreasing factor f(θ) are equal to 0.794 and 0.276 at a layer of continuum elements ahead of the crack tip, respectively. Based on the total concentration ($C=C_{L}+C_{T}$), the decreasing factor characterizes the effect of hydrogen on the ultimate embrittlement of this steel. Figure 16a shows the computed TSL of the fracture initiation for three initial hydrogen concentrations discussed above, and it can be calculated that hydrogen decreases the critical cohesive strength by 57% and 72% for $C_{L0}$ values of 3.5 and 8.5 ppm, respectively.

The criterion to measure the complete failure of cohesive element is the state when all two integration points of a cohesive element (COH2D4) fail. The numerical value corresponding to forward crack propagation is one element length when the cohesive stress reduces to a value of zero and its normalized separation is equal to one. Figure 16b describes the computational and experimental CTOD-R curves corresponding to previous set of parameters. In these curves, the results of the uncharged specimen from the simulation and the experiment are in good agreement with each other, which is the same as that presented in Figure 9b. However, for the hydrogen-charged specimens, the general trend of the simulation curve is the same as the experimental data, but not fully in agreement, the slightly higher curves indicate that the simulation significantly underestimated the experimentally measured crack propagation resistance. Indeed, according to the previous fracture micromechanism for the hydrogen-charged specimen, the fracture surfaces showed mixed ductile and brittle fracture surfaces, such as QC, IG and some evidence of small-scale MVC fracture. The microfracture characteristics showed that hydrogen embrittlement was a complex mechanism, and it is possible for more mechanisms to work together. The simulated model only adopts the HEDE model, but the HELP model and AIDE model are not taken into account in it.

## 6. Conclusions

In the present study, the fracture toughness test in a compact tension (CT) specimen made of low alloy 30CrMo steel and charged with hydrogen was analyzed, and then the coupled problems of stress and hydrogen diffusion with cohesive zone stress analysis was employed to simulate hydrogen-induced brittle fracture behavior using ABAQUS software. The major contents and conclusions of the present study are as follows:

1. From the fracture toughness test, the CTOD-R curves show a decreasing trend with the increase in the hydrogen charging time, which leads to the higher crack propagation and the lower crack growth resistance. This trend is also reflected in the change of fracture micromechanism: MVC is a dominant mode in an uncharged specimen, changing to QC fracture and to QC and IG fracture under hydrogen-charged condition.
2. In our simulation, the peak of hydrogen concentration (NNC) corresponds to the peak of hydrostatic stress, and is formed ahead of the crack tip. This result can explain the experimental phenomena well: hydrogen atoms diffusing ahead of the crack tip, thereby promoting the hydrogen embrittlement indexes.
3. The initial TSL parameters from the best fit to the load-displacement and J-R experimental curves without hydrogen were calibrated for the critical cohesive separation of 0.0393 mm and the cohesive strength of 2100 MPa. According to FEM and experimental curves, the initial normalized hydrogen concentrations corresponding to hydrogen-charged specimens for 12, 24 and 36 h were estimated 49.30, 77.46 and 119.72 NNC (corresponding to initial hydrogen concentration in lattice sites $C_{L0}$ of 3.5, 5.5 and 8.5 ppm), respectively.
4. The general trend of the computational CTOD-R curves is the same as that obtained from the experimental data but not fully in agreement, the computational CTOD values are slightly higher. Therefore, it is possible for more (HE) mechanisms to work together in order to predict the crack extension resistance correctly. The numerical model can provide design and prediction to calculate hydrogen-assisted fracture behavior prior to extensive laboratory testing, provided that the material properties and properly calibrated TSL parameters are known.

## Figures and Tables

**Table 1:** Chemical composition of the 30CrMo steel (wt.%).

C	Si	Mn	Cr	Ni	Mo	Cu
0.31	0.25	0.48	0.91	0.02	0.17	0.03

**Table 2:** Experimental true stress- plastic strain data for ABAQUS input analysis.

σ **(MPa)**	Plastic-Strain (mm/mm)
1067.2	0.003
1143.6	0.015
1192.9	0.04
1229	0.08
1240.9	0.1
1278.4	0.2
1329.8	0.5
1349.1	0.7
1363.8	0.9
1370	1.0

**Table 3:** Values of the parameters adopted in the model.

Parameter	Value	Source
Hydrogen solubility s	0.071 ppm mm N^−1/2^	
The universal gas constant R	8.314 J K^−1^ mol^−1^	[25]
Partial molar volume of hydrogen $V_{H}$	2.0 × 10^3^ mm^3^ mol^−^^1^	[34]
Hydrogen diffusion coefficient $D_{L}$	4.0 × 10^−5^ mm^2^ s^−^^1^	[23]
The Gibbs free energy $\Delta G_{g}^{0}$	30 KJ·mol^−^^1^	[37]
Temperature T	296 K	

**Table 4:** The TSL parameters for the uncharged specimen.

$\sigma_{\max}$ **(MPa)**	$\delta_{1}$ **(mm)**	$\delta_{2}$ **(mm)**	$\delta_{f}$ **(mm)**	$\sigma_{y}$ **(MPa)**
2100 (2 $\sigma_{y}$)	0.00197	0.0255	0.0393	1050

## Data Availability

Not applicable.
